# Conclusions Respecting Injections of Iodine in Ovarian Dropsy

**Published:** 1854-10

**Authors:** 


From the Monthly Jour Med.

*Conclusions respecting Injections of Iodine in Ovarian Dropsy.* By Professor SIMPSON.

Within the last year Dr. Simpson has, subsequently to tapping, injected into dropsical ovarian cysts the tincture of iodine in seven or eight cases. For this purpose he has employed the common tincture of iodine of the Edinburgh Pharmacopeia, undiluted. He has usually thrown into the cysts two or three ounces of the tincture. In some cases he has allowed a portion of the injected fluid to re-escape; in others, has retained the whole of it in the sac of the cyst that was tapped. From these cases he drew the following conclusions:

1. In none of the cases of ovarian dropsy, treated with iodine injections after tapping, has he yet seen any considerable amount of local pain follow the injection, with one exception; in most instances no pain at all is felt: and in none has constitutional irritation or fever ensued. In the one exceptional case considerable local irritation followed, and the pulse rose to 110; but the same phenomena occurred in the same patient after previous tappings without iodine being used.

2. While the practice seems thus so far perfectly safe in itself, it has by no means proved always as successful as in hydrocele in preventing a re-accumulation of the dropsical fluid; for in several instances the effusion into the sac seems to have gone on as rapidly as after a simple tapping without iodine injection.

3. But, in two or three of the cases, the iodine injection appears to have quite arrested for the time being the progress of the disease, and to have produced obliteration of the tapped cyst, as there is no sign whatever of any re-accumulation, though several months have now elapsed since the date of the operation.

Lastly. Accumulated experience will be required to point out more precisely the special varieties of ovarian dropsy most likely to benefit from iodine injections, the proper times of operating, the quantities of the tincture to be injected, and other correlative points. Perhaps the want of success in some cases has arisen from an insufficient quantity of iodine being used, and from the whole interior of the cyst not being touched by it. The greatest advantage would, of course, be expected from it in the rare form of unilocular ovarian cysts. In the common compound cyst, the largest or most preponderating cyst is usually alone opened in paracentesis; and, though it were obliterated, it would not necessarily prevent some of the other smaller cysts from afterwards enlarging and developing into the usual aggravated form of the disease.

